# Folate, Homocysteine, and Arsenic Metabolism in Arsenic-Exposed Individuals in Bangladesh

**DOI:** 10.1289/ehp.8084

**Published:** 2005-07-21

**Authors:** Mary V. Gamble, Xinhua Liu, Habibul Ahsan, J. Richard Pilsner, Vesna Ilievski, Vesna Slavkovich, Faruque Parvez, Diane Levy, Pam Factor-Litvak, Joseph H. Graziano

**Affiliations:** 1 Department of Environmental Health Sciences,; 2 Department of Biostatistics, and; 3 Department of Epidemiology, Mailman School of Public Health, Columbia University, New York, NY, USA; 4 Department of Pharmacology, College of Physicians and Surgeons, Columbia University, New York, NY, USA

**Keywords:** arsenic, arsenicosis, Bangladesh, creatinine, dimethylarsinic acid, folate, homocysteine, hyperhomocysteinemia, micronutrient deficiency, monomethylarsonic acid, one-carbon metabolism, *S*-adenosylmethionine, vitamin B_12_, well water

## Abstract

Chronic exposure to arsenic is occurring throughout South and East Asia due to groundwater contamination of well water. Variability in susceptibility to arsenic toxicity may be related to nutritional status. Arsenic is methylated to monomethylarsonic acid (MMA) and dimethylarsinic acid (DMA) via one-carbon metabolism, a biochemical pathway that is dependent on folate. The majority of one-carbon metabolism methylation reactions are devoted to biosynthesis of creatine, the precursor of creatinine. Our objectives of this cross-sectional study were to characterize the relationships among folate, cobalamin, homocysteine, and arsenic metabolism in Bangladeshi adults. Water arsenic, urinary arsenic, urinary creatinine, plasma folate, cobalamin, and homocysteine were assessed in 1,650 adults; urinary arsenic metabolites were analyzed for a subset of 300 individuals. The percentage of DMA in urine was positively associated with plasma folate (*r* = 0.14, *p* = 0.02) and negatively associated with total homocysteine (tHcys; *r* = −0.14, *p* = 0.01). Conversely, percent MMA was negatively associated with folate (*r* = −0.12, *p* = 0.04) and positively associated with tHcys (*r* = 0.21, *p* = 0.0002); percent inorganic arsenic (InAs) was negatively associated with folate (*r* = −0.12, *p* = 0.03). Urinary creatinine was positively correlated with percent DMA (*r* = 0.40 for males, *p* < 0.0001; 0.25 for females, *p* = 0.001), and with percent InAs (*r* = −0.45 for males, *p* < 0.0001; −0.20 for females, *p* = 0.01). Collectively, these data suggest that folate, tHcys, and other factors involved in one-carbon metabolism influence arsenic methylation. This may be particularly relevant in Bangladesh, where the prevalence of hyperhomocysteinemia is extremely high.

Arsenic that is naturally present in soil can be mobilized and transported, leading to increased concentrations of As in aquifers that are sources of drinking water (Harvey et al. 2002). The largest contemporary known mass exposure to As is occurring due to the consumption of tube-well water throughout the Ganges-Brahmaputra Delta in Bangladesh and India. In Bangladesh alone, this exposure is affecting approximately 25–30 million residents. Our survey of roughly 6,000 contiguous wells in Araihazar, Bangladesh, the region of interest in the current study, reported well-water As concentrations ranging from < 5 to 860 μg/L (van Geen et al. 2002). This range greatly exceeds the maximum contaminant level of 10 μg/L promulgated by the U.S. Environmental Protection Agency (EPA 2001) and the World Health Organization (WHO 2004), as well as the Bangladesh standard of 50 μg/L.

Individuals chronically exposed to As are at increased risk for various cancers, including cancers of the skin, bladder, lung, and liver. Chronic As exposure is also a risk factor for stroke (Chiou et al. 1997), ischemic heart disease (Hsueh et al. 1997), and neurologic consequences in adults and children (Wasserman et al. 2004). In addition, inorganic arsenic (InAs) has long been considered to be a teratogen in multiple mammalian species (Shalat et al. 1996; Wlodarczyk et al. 2001).

The biomethylation of InAs generates mono- and dimethyl arsenic species [mono-methylarsonic acid (MMA) and dimethylarsinic acid (DMA), respectively; Figure 1] which are more readily excreted than InAs (Vahter and Marafante 1987). Individuals whose urine contains relatively lower proportions of DMA have been reported to be at increased risk for skin and bladder cancers (Chen et al. 2003a, 2003b; Hsueh et al. 1997; Yu et al. 2000). Thus, methylation of InAs has traditionally been considered to be a detoxification pathway. However, a growing literature of experimental studies indicates that the trivalent methylated arsenic intermediates (MMA^III^ and DMA^III^) may be more toxic than InAs^III^ or any of the pentavalent intermediates (Ahmad et al. 1999; Lee et al. 1988; Nesnow et al. 2002; Styblo et al. 2002).

In humans, there is considerable inter-individual variability in the proportions of InAs, MMA, and DMA excreted in urine. InAs and MMA are enzymatically methylated via one-carbon metabolism, a biochemical pathway dependent on folate for recruitment of methyl groups. One-carbon metabolism also requires vitamins B_12_ (cobalamin) and B_6_ as cofactors (Figure 2). Not surprisingly, animal studies have suggested that folate nutritional status may influence As metabolism. For example, dietary folate deficiency (Spiegelstein et al. 2005) and/or dietary methyl donor deficiency significantly decreased total urinary As excretion, mainly due to lower DMA excretion; these diets also gave rise to increased retention of As in tissues, particularly in the liver and lungs (Tice et al. 1997; Vahter and Marafante 1987). These studies provided experimental evidence that the well-characterized nutritional regulation of one-carbon metabolism can influence As methylation and excretion.

The evidence for nutritional regulation of As methylation and excretion in humans is limited. In a study of 11 families in Chile, Chung et al. (2002) reported intrafamily associations in arsenic methylation. Although the correlation for InAs/(MMA+DMA) between father and mother was low (*r* = 0.18), adjustment for folate or homocysteine increased the correlation substantially (*r* = 0.33 and 0.55, respectively). The authors did not conclude that there was a significant effect of nutritional factors on methylation, but the data were highly suggestive (Chung et al. 2002). A case–control study in West Bengal, India, using dietary assessment, found a modest increase in risk for arsenicosis skin lesions for individuals within the lowest quintiles for dietary intake of animal protein, calcium, fiber, and folate (Mitra et al. 2004).

In the current study we tested the hypothesis that nutritional regulation of one-carbon metabolism, specifically folate nutritional status, contributes to the interindividual variability observed in InAs methylation. We conducted a cross-sectional study to assess the relationships among plasma total homocysteine (tHcys), total cysteine (Cys), folate, and cobalamin concentrations and As metabolism in adults residing in Araihazar, Bangladesh.

## Methods

The data presented are from the Nutritional Influences on Arsenic Toxicity (NIAT) study, an ongoing study on nutritional influences on arsenic metabolism. The NIAT study works in collaboration with a larger multidisciplinary program by health, earth, and social scientists from Columbia University and Bangladesh (the Columbia University Superfund Basic Research Program), the National Institute of Preventive and Social Medicine, and Dhaka University. The centerpiece of the public health research is a prospective cohort study, Health Effects of Arsenic Longitudinal Study (HEALS), of 12,000 adults exposed to a wide range of water As concentrations, from which the current sample is derived.

### Study region.

Bangladesh is a nation of roughly 124 million people inhabiting an area of 145,000 km^2^. It is divided into 64 districts, each of which is divided into 10–50 administrative units or *Thanas*. The study site is in Araihazar, one of 464 Thanas in Bangladesh, and is a 25-km^2^ region approximately 30 km east of Dhaka. The study site was chosen because *a*) it is known to have a wide range of As concentrations in the drinking water, permitting dose–response analyses and *b*) it is within a reasonable commuting distance from Dhaka. Socioeconomic data indicate that this region is not particularly poor by Bangladeshi standards.

### Eligibility criteria/participant recruitment/ethics.

The HEALS cohort study includes a random sample of 11,746 married men and women between 20 and 65 years of age who were recruited between September 2000 and May 2002 and are followed at 2-year intervals (Ahsan et al., in press). This study only included married couples to minimize the likelihood of loss to follow-up due to a change of residence after marriage. Centered around visits of one of the investigators (M.V.G.), a subset of 1,650 of these cohort study participants were consecutively enrolled in the present study. We further selected 300 of these 1,650 participants to measure urinary arsenic metabolites. This subset of participants was selected to be representative of the study population for total urinary As; the subset excluded those identified as being cobalamin deficient (plasma cobalamin concentrations < 185 pmol/L). Oral informed consent was obtained by our Bangladeshi field staff physicians, who read an institutional review board approved assent form to the study participants. Ethics approval was obtained from the Institutional Review Board of Columbia Presbyterian Medical Center and the Bangladesh Medical Research Council, and informed consent was obtained by our Bangladeshi field staff physicians.

### Plasma collection and handling.

We obtained plasma samples for tHcys, folate, and total cobalamin by venipuncture after the participant had been sitting for 10–15 min for an interview. Blood was collected into EDTA-containing tubes and immediately placed in IsoRack cool packs (Brinkmann Instruments, Westbury, NY) designed to maintain samples at 0°C for > 6 hr. Within 4 hr, samples were transported in hand-carried coolers containing additional ice packs to our local laboratory situated in our three-story field clinic in Araihazar. Samples were centrifuged at 4°C and plasma separated from the cells. Plasma was then stored in aliquots at −80°C and shipped frozen on dry ice to Columbia University for analysis.

### Measures of arsenic exposure.

We analyzed well-water arsenic concentrations as part of a comprehensive well survey before the onset of the cohort study. The actual well-water arsenic concentration was labeled onto each well, with signs indicating safety or danger, and many study participants subsequently switched wells to reduce exposure (van Geen et al. 2002); therefore, well-water arsenic did not always strictly represent current arsenic exposure. However, we do not believe that this led to significant exposure misclassification, as the duration of drinking water from the surveyed well greatly exceeded the duration of drinking water from an alternate well. In the analyses pertaining to folate and tHcys concentrations, any potential exposure misclassification would only bias to the null. Furthermore, we collected urine samples for urinary arsenic measurements on the same visit that the plasma samples were collected.

Well-water and urinary As provide two different estimates of As exposure. Although well-water As provides a direct measure of exposure that is uninfluenced by *in vivo* metabolism, it does not take into account the amount of water consumed or the accumulated body burden. Urinary As, traditionally considered a marker of recent exposure, may more closely reflect the body burden of As in a given individual because *a*) it receives contributions from tissue stores and *b*) it is more strongly associated with skin lesions than is well-water As (Ahsan et al. 2000). Urinary As concentrations are heavily influenced by variations in hydration status, as indicated by the association between total urinary As and urinary creatinine concentrations (*r* = 0.58, *p* < 0.0001, *N* = 1,650); thus, urinary creatinine must be included as a separate variable in the urinary As analyses. Because animal studies suggest that impaired one-carbon metabolism incurred by methyl-deficient diets decreases total urinary arsenic by up to 20–30% (Spiegelstein et al. 2003, 2005; Tice et al. 1997; Vahter and Marafante 1987), both urine As and well water As were included in the analyses.

### Total urinary As.

We measured total urinary As concentrations by graphite furnace atomic absorption spectrometry using the Analyst 600 graphite furnace system (Perkin Elmer, Shelton, CT) in the Columbia University Trace Metals Core Lab, essentially as previously described (Nixon et al. 1991). Our laboratory participates in a quality control program coordinated by Philippe Weber at the Quebec Toxicology Center in Quebec, Canada. During the course of this study, intra-class correlation coefficients between our laboratory’s values and samples calibrated at the Quebec laboratory were 0.99. Urinary creatinine concentrations were analyzed by a colorimetric Sigma Diagnostics Kit (Sigma, St. Louis, MO).

### Urinary arsenic metabolites.

Urinary arsenic metabolites were speciated for 300 participants using a method adapted from Heitkemper (Vela et al. 2001). This method employs HPLC separation of arsenobetaine (AsB), arsenocholine (AsC), arsenate, arsenite, MMA, and DMA, followed by detection by inductively-coupled mass spectrometry (ICP-MS). We calculated the percentages of InAs, MMA, and DMA after subtracting AsC and AsB from the total. In most cases, AsC and AsB were nondetectable.

### Plasma folate and cobalamin.

We analyzed folate and cobalamin in 1648 plasma samples by radioimmunoassay (Quantaphase II, Bio-Rad Laboratories, Richmond CA), as previously described (Gamble et al. 2005).

### Plasma homocysteine and cysteine concentrations.

We measured plasma tHcys and Cys concentrations in 1644 plasma samples by HPLC with fluorescence detection according to the method described by Pfeiffer et al. (1999), as described previously (Gamble et al. 2005). The within-day and between-day coefficients of variation for tHcys were 5% and 8%, respectively.

### Statistical analyses.

We calculated descriptive statistics for characteristics of the study sample separately by sex. Because we expected the distributions to be non-normal, sex differences in quantitative variables were tested using the Wilcoxon rank-sum test that requires no distribution assumption. We used chi-square tests to test for sex differences in categorical variables.

We used linear regression analyses to examine associations. Because As exposure might potentially influence plasma folate and tHcys concentrations, we explored the associations between these variables in the 1,650 samples. In sequential regression analyses on the subset of 300 participants with urinary arsenic metabolite data, we examined determinants of percentages of InAs (%InAs), MMA(%MMA), and DMA (%DMA).

Because tHcys, folate, and cobalamin in plasma, and water arsenic, urine arsenic, and urine creatinine have skewed distributions, we used log-transformation to achieve approximately symmetric distributions. In preliminary analyses, water arsenic, age, sex, cigarette smoking, and betelnut use were found to be associated with urinary arsenic metabolites and were therefore included in our regression models as potential confounding variables.

## Results

### Characteristics of the population.

The general characteristics of the NIAT study sample have been previously described in detail (Gamble et al. 2005). Out of 1,650 participants, 973 were women and 677 were men. The mean ages for women and men were 34.6 ± 8.8 and 42.2 ± 9.8 years, and body mass indices were 20.2 ± 3.2 and 19.4 ± 3.0, respectively. Betelnut use was practiced by 30% of women and 40% of men, whereas 6% of women and 76% of men smoked cigarettes.

### tHcys, folate, and cobalamin findings.

We recently reported a high prevalence of hyper-homocysteinemia, particularly among males (63% ≥ 11.4 μM) in this study population (Gamble et al. 2005). Folate and cobalamin nutritional status accounted for 15% and 5%, respectively, of the variability in tHcys.

### Arsenic exposure.

As is shown in Table 1, urinary As concentrations, expressed in micrograms per liter, did not differ between males and females. However, when adjusted for urinary creatinine, as is routinely done to correct for the effects of hydration, males had significantly lower As concentrations than females. This divergence is attributable to significant sex differences in urinary creatinine, which is related to lean body mass (Schutte et al. 1981). Well-water As concentrations ranged from 0.1 μg/L to 650 μg/L, with 82% of wells having concentrations > 10 μg/L and 63% > 50 μg/L.

### Influence of arsenic exposure on folate, tHcys and Cys.

**Figure 2:**
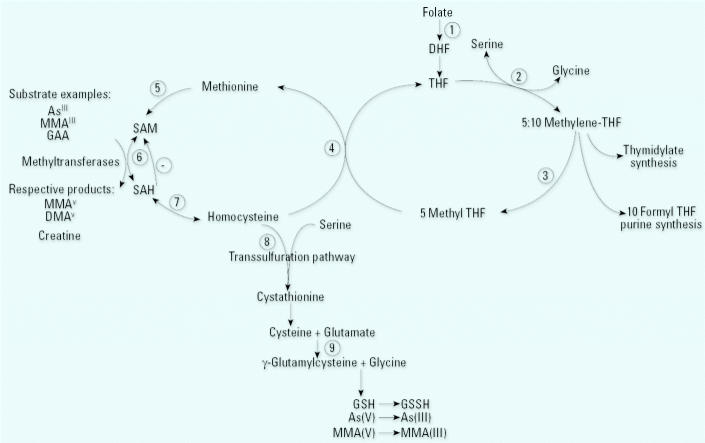
Overview of one-carbon metabolism. 1. Dietary folates are reduced to dihydrofolate (DHF) and tetrahydrofolate (THF) by dihydrofolate reductase. 2. The β-carbon of serine is transferred to THF by serine hydroxymethyltransferase, forming 5,10-methenyl-THF and glycine. 3. At a major branch point between transmethylation reactions and nucleotide biosynthesis, 5,10-methenyl-THF can be reduced to 5,10-methylene-THF and further reduced to 5-methyl-THF by 5,10-methylene-THF reductase. 4. In a reaction catalyzed by the vitamin B_12_-containing enzyme, methionine synthetase, the methyl group of 5-methyl-THF is transferred to homocysteine, generating methionine and regenerating THF. 5. Methionine adenosyl-transferase activates methionine to form *S*-adenosylmethionine (SAM). 6. SAM serves as a universal methyl donor for numerous acceptors, including predominantly guanidinoacetate (GAA), but also DNA, arsenic, and others, in reactions that involve a number of methyltransferases. 7. The by-product of these methylation reactions, *S*-adenosylhomocysteine (SAH), is hydrolyzed to generate homocysteine. SAH is a potent inhibitor of most SAM-dependent methylations. 8. Homocysteine is either used to regenerate methionine or is directed to the transsulfuration pathway through which it is ultimately catabolized. 9. The transsulfuration pathway is also responsible for glutathione (GSH) biosynthesis.

Among the entire sample (*n* = 1,650), water arsenic concentrations were significantly associated with plasma folate (*r* = −0.13, *p* < 0.0001), tHcys (*r* = 0.05, *p* = 0.03), and Cys (*r* = 0.13, *p* < 0.0001). Although total urinary arsenic (micrograms per gram creatinine) was also negatively associated with plasma folate (*r* = −0.15, *p* < 0.0001), it was not associated with tHcys or Cys (*r* = −0.03, *p* = 0.29; *r* = −0.03, *p* = 0.23 ). Folate deficiency may influence urinary creatinine concentrations because the synthesis of creatine, the precursor of creatinine, accounts for approximately 75% of folate- and *S*-adeno-sylmethionine (SAM)-dependent transmethylation reactions (Figure 2, reaction 6; Mudd et al. 1975). Thus, we sought to rule out the possibility that the inverse association between plasma folate and urinary As per gram creatinine might be due to an association between plasma folate and urinary creatinine. Indeed, plasma folate concentrations were positively correlated with urinary creatinine for males (*r* = 0.083, *p* = 0.0308) (Gamble et al. 2005). Urinary arsenic concentrations (micrograms per liter) were negatively associated with plasma folate (*r* = −0.13, *p* < 0.0001), even when not adjusted for urinary creatinine.

### tHcys, folate, cobalamin, and urinary arsenic metabolites.

**Figure 3:**
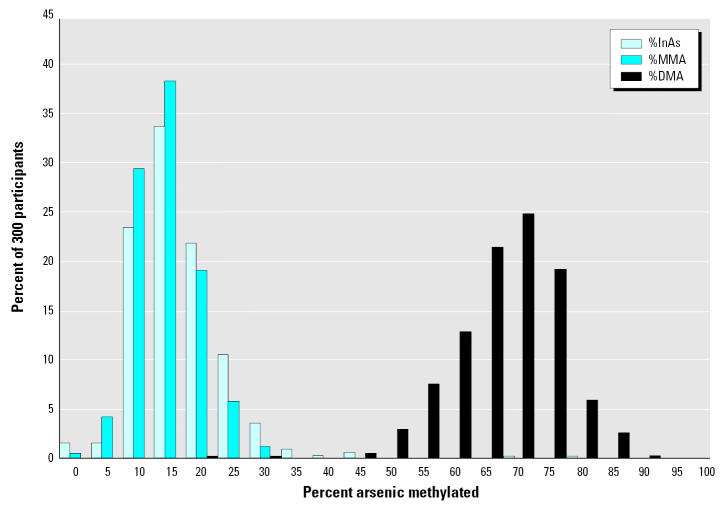
Frequency distribution for As metabolites. Interindividual variability across 300 participants for arsenic metabolites in urine.

Urinary As metabolites measured in a subset of 300 study participants showed a wide interindividual variability in arsenic methylation capacity. For example, the percentage of total urinary As present as DMA (%DMA) ranged from 20 to 90%, with the majority falling between 50 and 80% (Figure 3). In addition, methylation capacity differed by sex: on average, females had a higher %DMA than males (72.2 ± 10.4 vs. 69.7 ± 7.7 %DMA, respectively, *p* = 0.0012) and a lower %MMA (11.5 ± 4.8 vs. 15.5 ± 5.2 %MMA, respectively, *p* < 0.0001). The %InAs did not differ by sex (16.3 ± 10.1 vs. 14.7 ± 5.5, *p* = 0.60). The sex differences persisted in regression analyses for %DMA and %MMA after adjustment for other covariates including age and water arsenic (data not shown). Although age was not significantly associated with %DMA, it was positively associated with %MMA and negatively associated with %InAs (*r* = 0.29, *p* < 0.0001, and *r* = −0.24, *p* < 0.0001, respectively). Although only 12 of these 300 participants had arsenic-related skin lesions, the presence of skin lesions was associated with higher %MMA (18.5 ± 5.2 vs. 13.1 ± 5.3, *p* = 0.0016) and with lower %DMA (66.6 ± 9.1 vs. 71.3 ± 9.3, *p* = 0.0518). These findings have recently been confirmed in a much larger sample of the HEALS cohort study (data not shown).

As hypothesized, %DMA was positively associated with plasma folate and negatively associated with plasma tHcys (Table 2). Conversely, %MMA was negatively associated with plasma folate and positively associated with tHcys. Although %InAs was also negatively associated with folate, the association with tHcys was not significant. In addition, plasma Cys concentrations were negatively correlated with %InAs and positively correlated with %MMA. Plasma cobalamin concentrations were not correlated with arsenic metabolites.

Due to the clear biochemical link between creatine biosynthesis and SAM-dependent transmethylation reactions (Figure 2, reaction 6), the expression of urinary As per gram creatinine might potentially confound the associations with arsenic metabolites. To explore this possibility, we examined the association between urinary creatinine and urinary arsenic metabolites. We observed that urinary creatinine was strongly and positively associated with %DMA for both males and females and negatively associated with %InAs and with %MMA for females (Table 3). These associations remained highly significant after controlling for other covariates including body weight, age, and water arsenic. Furthermore, the associations between As metabolites and folate or tHcys became nonsignificant when urinary creatinine was included in regression analyses.

We previously reported that cigarette smoking and betelnut use are both negative predictors of plasma folate and positive predictors of tHcys (Gamble et al. 2005). Betelnut use also proved to be a significant predictor of %MMA, even after adjusting for covariates including sex, cigarette smoking, water arsenic, urinary creatinine, and plasma folate (*p* = 0.03).

## Discussion

Our previous observation of a high prevalence of hyperhomocysteinemia in Araihazar, Bangladesh (Gamble et al. 2005) afforded us a suitable setting in which to assess the potential impact of hyperhomocysteinemia and folate deficiency on arsenic metabolism in a human population. Although cobalamin concentrations were not significantly associated with arsenic metabolism, this finding was not unexpected because *a*) cobalamin deficiency is relatively rare in this region, where vegetarianism is uncommon, and *b*) cobalamin-deficient participants were excluded from the study on arsenic metabolites. Thus, the results of this study do not rule out the possibility that cobalamin deficiency may influence arsenic methylation. In general, although the effect sizes were relatively small, the associations between As methylation and folate and tHcys were as predicted; however, a few points are of particular interest. First, the observation that folate is negatively (and equally) associated with both %InAs and %MMA implies that adequate folate nutritional status is required for both the first and the second methylation steps. This may be of clinical relevance because a growing body of evidence links increased risk for adverse health outcomes to higher %MMA and reduced risk with higher %DMA in urine (Chen et al. 2003a, 2003b; Hsueh et al. 1997; Yu et al. 2000).

A second finding of interest is our observation that tHcys is not associated with %InAs but is positively associated with %MMA (Table 2). In one-carbon metabolism, all SAM-dependent methylation reactions yield the methylated product and *S*-adenosylhomocysteine (SAH; Figure 2, reaction 6). SAH is then hydrolyzed to homocysteine in a reaction that is readily reversible. Plasma SAH levels increase linearly with even mild elevations in homocysteine levels (Yi et al. 2000). This is of particular relevance because SAH is a potent product inhibitor of most transmethylation reactions (Yi et al. 2000), including those of arsenic (De Kimpe et al. 1999). SAH binds tightly to methyltransferases and is only removed if the pathway is pulled forward by downstream removal of tHcys, as might be achieved with folate supplementation (Figure 2, reaction 4). SAH is actually more crucial than SAM in regulating methylation reactions (Yi et al. 2000). Thus, it is possible that the positive association between tHcys and %MMA and negative association with %DMA may reflect inhibition of the second methylation step by SAH. It is not entirely clear why tHcys is not also positively associated with %InAs. Perhaps because SAH binds to the arsenic methyltransferase during the first methylation step, it subsequently inhibits the second methylation step. The potential for folate supplementation to reverse hyperhomocysteinemia and thereby facilitate arsenic methylation is currently being tested in a placebo-controlled double-blind folate supplementation trial to folate deficient participants.

The amino acid Cys is produced from Hcys as an intermediate in glutathione biosynthesis (Figure 2, reaction 8). Consequently, Cys concentrations in plasma are positively correlated with both tHcys and glutathione (GSH). Cys has recently been reported to play a role in redox cycling (Jones et al. 2004). Like glutathione, Cys is capable of reducing pentavalent As^V^ to As^III^ *in vitro* (Celkova et al. 1996), and this reduction is a prerequisite to methylation. The observation that plasma Cys concentrations are negatively associated with %InAs and positively associated with %MMA suggests that this capacity may be physiologically relevant. However, we cannot rule out the possibility that the observed associations are simply due to a positive association between Cys and plasma GSH. The complete lack of an association with %DMA suggests that Cys (or possibly GSH) does not similarly reduce MMA^V^ to MMA^III^ to facilitate the second methylation step.

A number of noteworthy observations regarding urinary creatinine arose from this study. The formation of creatine from methylation of guanidinoacetate has been estimated to account for approximately 75% of transmethylation reactions (Mudd and Poole 1975). Creatine is a precursor of creatinine, and both are synthesized and circulate at concentrations proportional to muscle mass, which is generally greater in males than females. Consequently, urinary creatinine is also greater for males than females (Barr et al. 2005; Gamble and Liu 2005). Because creatinine is excreted at a relatively constant rate throughout the course of the day, it is commonly used as a correction factor to adjust for variations in urine concentration. It is important to note, however, that this adjustment introduces an artificial sex difference such that, using total urinary arsenic/g creatinine as the exposure variable, males may artificially appear to have a lower exposure than females. This finding has particular relevance to the field of As toxicology because it has been reported that males may be more susceptible than females to arsenic toxicity (Chen et al. 2003a; Kristiansen et al. 1997) for two reasons. First, creatinine adjustment causes males to appear to have a lower exposure than females (Table 1). Second, although speculative, it is also possible that the increased susceptibility among males is related to the fact that males place higher demands on one-carbon metabolism due to their greater muscle mass and associated demands for creatine formation and resultant higher tHcys (Brattstrom et al. 1994).

The associations between the methylation of As and urinary creatinine are quite remarkable and intriguing. In this study, urinary creatinine was the strongest predictor by far of arsenic methylation. Particularly for males, the effect sizes were more than double those of any of the nutritional variables. There are a number of plausible mechanisms which could explain these associations: *a*) for reasons described above, urinary creatinine may serve as a proxy for one-carbon “metabolic rate”; *b*) urinary creatinine is a known proxy for muscle mass, which could have some unknown impact on As methylation; *c*) urinary creatinine is influenced by dietary protein intake, which influences one-carbon metabolism in-so-far as it provides a source of methionine; and *d*) urinary creatinine is influenced by renal function, which, for reasons that remain unclear, is a primary determinant of tHcys concentrations (Arnadottir et al. 1996; Wollesen et al. 1999). Any of these mechanisms acting either independently or in concert may play a role in determining what fraction of InAs is methylated.

Like cigarette smoking and betelnut use (Gamble et al. 2005), total urinary As and well-water As were found to be negatively associated with plasma folate. Because folate is highly prone to oxidative degradation, and because there is a substantial basic literature indicating that exposure to As induces oxidative stress, it is possible that this observation is attributable to As-induced oxidative degradation of folate. The positive associations between well-water As and tHcys and Cys likely follow from their metabolic interrelations with folate.

Alternatively, it has been postulated that increased demands on one-carbon metabolism for the methylation of As in those chronically exposed to As-contaminated drinking water may deplete the methyl donor pool (Mass and Wang 1997). We have not measured the hepatic methyl donor pool, nor is it entirely clear from our understanding of folate and one-carbon metabolism what effect depletion of the methyl donor pool would have on plasma folate concentrations. If one were to assume that methyl depletion would increase folate turnover, then plasma folate concentrations would be reduced. This is another potential explanation for the observation that well water As and urinary As were negatively associated with plasma folate, but determination of the mechanism underlying this observation will require additional study.

In conclusion, the results of this cross-sectional study suggest that adequate folate nutritional status facilitates both the first and second methylation steps, resulting in excretion of lower proportions of InAs and MMA and higher DMA. Conversely, individuals with hyperhomocysteinemia have a reduced ability to methylate MMA to generate DMA. As evidence has accrued linking increased risk for various adverse health outcomes to higher %MMA and lower %DMA in urine, it appears that hyperhomocysteinemia may represent a modifiable risk factor for arsenic toxicity. This may be particularly important in Bangladesh, where the incidence of hyperhomocysteinemia is extremely high (Gamble et al. 2005). Elucidation of the mechanism(s) underlying the relationship between urinary creatinine and arsenic methylation deserves further investigation.

## Figures and Tables

**Figure 1:**
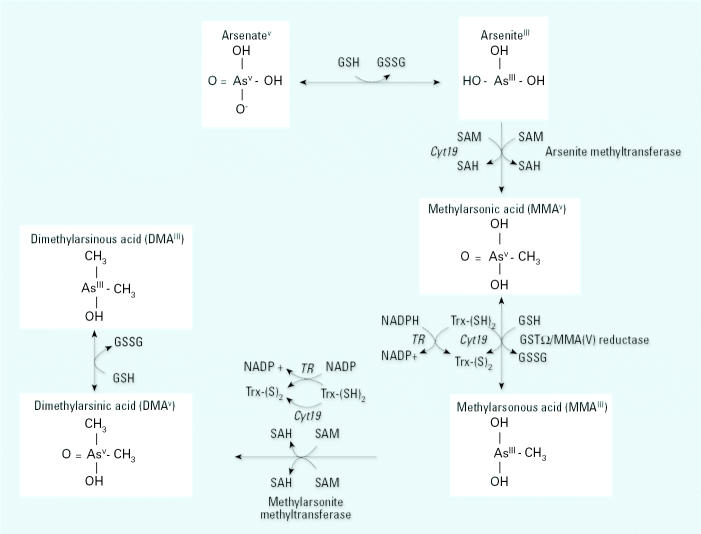
Arsenic metabolic pathway. Arsenate is reduced to arsenite in a reaction thought to be dependent on GSH or other endogenous reductants. Abbreviations: GSH, glutathione; GSSG, glutathione disulfide; GST, glutathione-*S*-transferase; SAH, *S*-adenosylhomocysteine; SAM, *S*-adenosylmethionine; TR, thioredoxin reductase. Arsenite then undergoes an oxidative methylation, with SAM as the methyl donor, forming MMA^V^ and SAH. MMA^V^ is reduced to MMA^III^ before a subsequent oxidative methylation step yielding DMA^V^ and SAH. Little is known regarding *in vivo* reduction of DMA^V^ to DMA^III^. Enzymes capable of catalyzing the illustrated reactions include *Cyt19* (Lin et al. 2002), arsenite methyltransferase and methy-larsonite methyltransferase (two activities of one enzyme) (Zakharyan et al. 1995), and MMA^V^ reductase (also known as GST-Ω) (Zakharyan et al. 2001).

**Table 1:** Sex differences in urinary creatinine and urinary arsenic when expressed per gram creatinine (mean ± SD).

	Females (*n* = 961)	Males (*n* = 675)	*p*-Value
Urinary arsenic (μg/L)	134 ± 120	133 ± 137	0.26
Urinary creatinine (mg/dL)	57 ± 41	70 ± 53	< 0.0001
Arsenic/g creatinine	284 ± 226	194 ± 179	< 0.0001

**Table 2:** Spearman correlation coefficients for As metabolites versus plasma folate, tHcys, and cobalamin for 300 participants.

	%InAs	%MMA	%DMA
tHcys (μM)	0.06	0.21*^#^*	−0.14**
Cysteine (μM)	−0.11*	0.16**	0.01
Folate (nM)	−0.12*	−0.12*	0.14*
Cobalamin (pM)	−0.06	−0.002	0.04

* *p* < 0.05.

** *p* < 0.01.

# *p* < 0.001.

**Table 3:** Spearman correlation coefficients for As metabolites versus urinary creatinine.

	%InAs	%MMA	%DMA
Males (*n* = 136)	−0.45*^##^*	−0.13	0.40*^##^*
Females (*n* = 164)	−0.20**^a^	−0.16*	0.25**
Total (*n* = 300)	−0.32*^##^*	−0.09	0.30*^##^*

a Correlation between urinary creatinine and %InAs differ by sex (*p* = 0.014).

* *p* < 0.05.

** *p* < 0.01.

## *p* < 0.0001.

## References

[b1-ehp0113-001683] Ahmad S, Anderson WL, Kitchin KT (1999). Dimethylarsinic acid effects on DNA damage and oxidative stress related biochemical parameters in B6C3F1 mice. Cancer Lett.

[b2-ehp0113-001683] Ahsan H Chen Y Parvez F Zablotska L Argos L Hussain A2005. Health Effects of Arsenic Longitudinal Study (HEALS) a multidisciplinary epidemiologic investigation. J Expos Anal Environ Epidemiol 10.1038/sj.jea.7500449. 10.1038/sj.jea.7500449 16160703

[b3-ehp0113-001683] Ahsan H, Perrin M, Rahman A, Parvez F, Stute M, Zheng Y (2000). Associations between drinking water and urinary arsenic levels and skin lesions in Bangladesh. J Occup Environ Med.

[b4-ehp0113-001683] Arnadottir M, Hultberg B, Nilsson-Ehle P, Thysell H (1996). The effect of reduced glomerular filtration rate on plasma total homocysteine concentration. Scand J Clin Lab Invest.

[b5-ehp0113-001683] Barr DB, Wilder LC, Caudill SP, Gonzalez AJ, Needham LL, Pirkle JL (2005). Urinary creatinine concentrations in the U.S. population: implications for urinary biologic monitoring measurements. Environ Health Perspect.

[b6-ehp0113-001683] Brattstrom L, Lindgren A, Israelsson B, Andersson A, Hultberg B (1994). Homocysteine and cysteine: determinants of plasma levels in middle-aged and elderly subjects. J Intern Med.

[b7-ehp0113-001683] Celkova A, Kubova J, Stresko V (1996). Determination of arsenic in geological samples by HG AAS. Anal Bioanal Chem.

[b8-ehp0113-001683] Chen YC, Guo YL, Su HJ, Hsueh YM, Smith TJ, Ryan LM (2003a). Arsenic methylation and skin cancer risk in southwestern Taiwan. J Occup Environ Med.

[b9-ehp0113-001683] Chen YC, Su HJ, Guo YL, Hsueh YM, Smith TJ, Ryan LM (2003b). Arsenic methylation and bladder cancer risk in Taiwan. Cancer Causes Control.

[b10-ehp0113-001683] Chiou HY, Huang WI, Su CL, Chang SF, Hsu YH, Chen CJ (1997). Dose-response relationship between prevalence of cerebrovascular disease and ingested inorganic arsenic. Stroke.

[b11-ehp0113-001683] Chung JS, Kalman DA, Moore LE, Kosnett MJ, Arroyo AP, Beeris M (2002). Family correlations of arsenic methylation patterns in children and parents exposed to high concentrations of arsenic in drinking water. Environ Health Perspect.

[b12-ehp0113-001683] De Kimpe J, Cornelis R, Vanholder R (1999). In vitro methylation of arsenite by rabbit liver cytosol: effect of metal ions, metal chelating agents, methyltransferase inhibitors and uremic toxins. Drug Chem Toxicol.

[b13-ehp0113-001683] Gamble MV, Ahsan H, Liu X, Factor-Litvak P, Ilievski V, Slavkovich V (2005). Folate and cobalamin deficiencies and hyperhomocysteinemia in Bangladesh. Am J Clin Nutr.

[b14-ehp0113-001683] Gamble MV, Liu X (2005). Urinary creatinine concentrations in the U.S. population: implications for urinary biologic monitoring measurements [Letter]. Environ Health Perspect.

[b15-ehp0113-001683] Harvey CF, Swartz CH, Badruzzaman AB, Keon-Blute N, Yu W, Ali MA (2002). Arsenic mobility and groundwater extraction in Bangladesh. Science.

[b16-ehp0113-001683] Hsueh YM, Chiou HY, Huang YL, Wu WL, Huang CC, Yang MH (1997). Serum beta-carotene level, arsenic methylation capability, and incidence of skin cancer. Cancer Epidemiol Biomarkers Prev.

[b17-ehp0113-001683] Jones DP, Go YM, Anderson CL, Ziegler TR, Kinkade JM, Kirlin WG (2004). Cysteine/cystine couple is a newly recognized node in the circuitry for biologic redox signaling and control. FASEB J.

[b18-ehp0113-001683] Kristiansen J, Christensen JM, Iversen BS, Sabbioni E (1997). Toxic trace element reference levels in blood and urine: influence of gender and lifestyle factors. Sci Total Environ.

[b19-ehp0113-001683] Lee TC, Tanaka N, Lamb PW, Gilmer TM, Barrett JC (1988). Induction of gene amplification by arsenic. Science.

[b20-ehp0113-001683] Lin S, Shi Q, Nix FB, Styblo M, Beck MA, Herbin-Davis KM (2002). A novel *S*-adenosyl-l-methionine: arsenic(III) methyltransferase from rat liver cytosol. J Biol Chem.

[b21-ehp0113-001683] Mass MJ, Wang L (1997). Arsenic alters cytosine methylation patterns of the promoter of the tumor suppressor gene p53 in human lung cells: a model for a mechanism of carcinogenesis. Mutat Res.

[b22-ehp0113-001683] Mitra SR, Mazumder DN, Basu A, Block G, Haque R, Samanta S (2004). Nutritional factors and susceptibility to arsenic-caused skin lesions in West Bengal, India. Environ Health Perspect.

[b23-ehp0113-001683] Mudd SH, Poole JR (1975). Labile methyl balances for normal humans on various dietary regimens. Metabolism.

[b24-ehp0113-001683] Nesnow S, Roop BC, Lambert G, Kadiiska M, Mason RP, Cullen WR (2002). DNA damage induced by methylated trivalent arsenicals is mediated by reactive oxygen species. Chem Res Toxicol.

[b25-ehp0113-001683] Nixon D, Mussmann G, Eckdahl S, Moyer T (1991). Total arsenic in urine: palladium-persulfate vs nickel as a matrix modifier for graphite furnace atomic absorption spectrophotometry. Clin Chem.

[b26-ehp0113-001683] Pfeiffer CM, Huff DL, Gunter EW (1999). Rapid and accurate HPLC assay for plasma total homocysteine and cysteine in a clinical laboratory setting. Clin Chem.

[b27-ehp0113-001683] Schutte JE, Longhurst JC, Gaffney FA, Bastian BC, Blomqvist CG (1981). Total plasma creatinine: an accurate measure of total striated muscle mass. J Appl Physiol.

[b28-ehp0113-001683] Shalat SL, Walker DB, Finnell RH (1996). Role of arsenic as a reproductive toxin with particular attention to neural tube defects. J Toxicol Environ Health.

[b29-ehp0113-001683] Spiegelstein O, Lu X, Le XC, Troen A, Selhub J, Melnyk S (2003). Effects of dietary folate intake and folate binding protein-1 (Folbp1) on urinary speciation of sodium arsenate in mice. Toxicol Lett.

[b30-ehp0113-001683] Spiegelstein O, Lu X, Le CX, Troen A, Selhub J, Melnyk S (2005). Effects of dietary folate intake and folate binding protein-2 (Folbp2) on urinary speciation of sodium arsenate in mice. Environ Toxicol Pharmacol.

[b31-ehp0113-001683] Styblo M, Drobna Z, Jaspers I, Lin S, Thomas DJ (2002). The role of biomethylation in toxicity and carcinogenicity of arsenic: a research update. Environ Health Perspect.

[b32-ehp0113-001683] Tice RR, Yager JW, Andrews P, Crecelius E (1997). Effect of hepatic methyl donor status on urinary excretion and DNA damage in B6C3F1 mice treated with sodium arsenite. Mutat Res.

[b33-ehp0113-001683] U.S. EPA 2001. National Primary Drinking Water Regulations; Arsenic and Clarifications to Compliance and New Source Contaminants Monitoring; Final Rule. Fed Reg 66(14):6975–7066. Available: http://www.epa.gov/safewater/ars/arsenic_finalrule.pdf [accessed 24 October 2005].

[b34-ehp0113-001683] Vahter M, Marafante E (1987). Effects of low dietary intake of methionine, choline or proteins on the biotransformation of arsenite in the rabbit. Toxicol Lett.

[b35-ehp0113-001683] van Geen A, Ahsan H, Horneman AH, Dhar RK, Zheng Y, Hussain I (2002). Promotion of well-switching to mitigate the current arsenic crisis in Bangladesh. Bull WHO.

[b36-ehp0113-001683] Vela NP, Heitkemper DT, Stewart KR (2001). Arsenic extraction and speciation in carrots using accelerated solvent extraction, liquid chromatography and plasma mass spectrometry. Analyst.

[b37-ehp0113-001683] Wasserman GA, Liu X, Parvez F, Ahsan H, Factor-Litvak P, van Geen A (2004). Water arsenic exposure and children’s intellectual function in Araihazar, Bangladesh. Environ Health Perspect.

[b38-ehp0113-001683] WHO 2004. Guidelines for Drinking Water Quality. 3rd Ed, Vol. 1, Recommendations. Geneva:World Health Organization.

[b39-ehp0113-001683] Wlodarczyk B, Spiegelstein O, Gelineau-van WJ, Vorce RL, Lu X, Le CX (2001). Arsenic-induced congenital malformations in genetically susceptible folate binding protein-2 knockout mice. Toxicol Appl Pharmacol.

[b40-ehp0113-001683] Wollesen F, Brattstrom L, Refsum H, Ueland PM, Berglund L, Berne C (1999). Plasma total homocysteine and cysteine in relation to glomerular filtration rate in diabetes mellitus. Kidney Int.

[b41-ehp0113-001683] Yi P, Melnyk S, Pogribna M, Pogribny IP, Hine RJ, James SJ (2000). Increase in plasma homocysteine associated with parallel increases in plasma *S*-adenosylhomocysteine and lymphocyte DNA hypomethylation. J Biol Chem.

[b42-ehp0113-001683] Yu RC, Hsu KH, Chen CJ, Froines JR (2000). Arsenic methylation capacity and skin cancer. Cancer Epidemiol Biomarkers Prev.

[b43-ehp0113-001683] Zakharyan RA, Sampayo-Reyes A, Healy SM, Tsaprailis G, Board PG, Liebler DC (2001). Human monomethylarsonic acid (MMA(V)) reductase is a member of the glutathione-*S*-transferase superfamily. Chem Res Toxicol.

[b44-ehp0113-001683] Zakharyan R, Wu Y, Bogdan GM, Aposhian HV (1995). Enzymatic methylation of arsenic compounds: assay, partial purification, and properties of arsenite methyltransferase and monomethylarsonic acid methyltransferase of rabbit liver. Chem Res Toxicol.

